# Role of E6 in Maintaining the Basal Cell Reservoir during Productive Papillomavirus Infection

**DOI:** 10.1128/jvi.01181-21

**Published:** 2022-03-09

**Authors:** Taylor Saunders-Wood, Nagayasu Egawa, Ke Zheng, Alberto Giaretta, Heather M. Griffin, John Doorbar

**Affiliations:** a Department of Pathology, University of Cambridge, Cambridge, United Kingdom; b Department of Information Engineering, University of Padova, Padua, Italy; International Centre for Genetic Engineering and Biotechnology

**Keywords:** E6, papillomavirus, epithelial homeostasis, lesion formation, lesion maintenance, productive viral life cycle

## Abstract

Papillomaviruses exclusively infect stratified epithelial tissues and cause chronic infections. To achieve this, infected cells must remain in the epithelial basal layer alongside their uninfected neighbors for years or even decades. To examine how papillomaviruses achieve this, we used the *in vivo* MmuPV1 (Mus musculus papillomavirus 1) model of lesion formation and persistence. During early lesion formation, an increased cell density in the basal layer, as well as a delay in the infected cells’ commitment to differentiation, was apparent in cells expressing MmuPV1 E6/E7 RNA. Using cell culture models, keratinocytes exogenously expressing MmuPV1 E6, but not E7, recapitulated this delay in differentiation postconfluence and also grew to a significantly higher density. Cell competition assays further showed that MmuPV1 E6 expression led to a preferential persistence of the cell in the first layer, with control cells accumulating almost exclusively in the second layer. Interestingly, the disruption of MmuPV1 E6 binding to MAML1 protein abrogated these phenotypes. This suggests that the interaction between MAML1 and E6 is necessary for the lower (basal)-layer persistence of MmuPV1 E6-expressing cells. Our results indicate a role for E6 in lesion establishment by facilitating the persistence of infected cells in the epithelial basal layer, a mechanism that is most likely shared by other papillomavirus types. Interruption of this interaction is predicted to impede persistent papillomavirus infection and consequently provides a novel treatment target.

**IMPORTANCE** Persistent infection with high-risk HPV types can lead to development of HPV-associated cancers, and persistent low-risk HPV infection causes problematic diseases, such as recurrent respiratory papillomatosis. The management and treatment of these conditions pose a considerable economic burden. Maintaining a reservoir of infected cells in the basal layer of the epithelium is critical for the persistence of infection in the host, and our studies using the mouse papillomavirus model suggest that E6 gene expression leads to the preferential persistence of epithelial cells in the lower layers during stratification. The E6 interaction with MAML1, a component of the Notch pathway, is required for this phenotype and is linked to E6 effects on cell density and differentiation. These observations are likely to reflect a common E6 role that is preserved among papillomaviruses and provide us with a novel therapeutic target for the treatment of recalcitrant lesions.

## INTRODUCTION

Papillomaviruses (PVs) are small, nonenveloped, double-stranded DNA tumor viruses that infect more than 80 different species. Over 405 papillomavirus genomes are currently listed in the Papillomavirus Episteme (http://pave.niaid.nih.gov), of which 198 infect humans. PVs have a common genome organization and encode “core” proteins required for viral genome replication and packaging (i.e., E1, E2, L1, and L2), along with a number of more divergent “accessory proteins,” such as E6 and E7, which modify the infected cell to allow replication and persistence. In high-risk PVs, E6 and E7 are considered oncogenes and are important in the development of PV-associated cancers. The majority of PVs are, however, classified as low risk and are associated only with benign papillomas or inapparent infections (1). PV evolution and diversification has been impacted by the colonization of specific epithelial niches, with coevolution and niche adaptation allowing PVs to develop their remarkable species and tissue specificity (2). Although papillomavirus protein functions may vary between PV species and types, as a group they share common life cycle strategies, including the need to persist in the epithelial basal layer following infection. In this context, animal models of infection have proven useful in establishing many of the basic principles that control how papillomavirus lesions form and how productive infection is regulated by viral gene products.

Until recently, the field has lacked a PV that can infect and be propagated in laboratory mice. MmuPV1 (Mus musculus papillomavirus 1 [originally MusPV]) (3), a member of the pipapillomavirus genus, was isolated in 2011 from cutaneous lesions (4), with subsequent research demonstrating an additional ability to infect a range of mucosal sites (5–7). Although generally considered a model of human betapapillomavirus infections, it has also been used to study PV carcinogenesis at the female reproductive tract, a site which is targeted in humans by the high-risk alphapapillomaviruses (8). The availability of MmuPV1 provides an opportunity to model many fundamental principles of PV infection and to develop a broader understanding of PV biology and disease pathogenesis (9–13).

Human papillomaviruses (HPVs) are split into five genera based on nucleic acid sequence divergence, with high-risk alpha PVs causing a range of human cancers (14). Low-risk alphapapillomaviruses generally cause benign warts, with beta and gamma types typically associated with only persistent asymptomatic infections in immunocompetent individuals (1). Although beta PVs are part of the normal commensal microbial flora (15), they can cause cutaneous squamous cell carcinoma (cSCC) in patients suffering from epidermodysplasia verruciformis (EV) (16, 17). PV infection typically occurs following a microwound, which allows virus particles to access the mitotically active basal cells on the basal lamina (reviewed in reference 18), which are responsible for the maintenance and replenishment of all layers of the skin.

To maintain epithelial homeostasis, the loss of cells from the basal layer must be precisely matched by basal cell division, which is considered to be mediated by discrete groups of slow-cycling stem cells that maintain progeny transit-amplifying cells, to produce epidermal proliferative units (EPUs) (19, 20). Lineage tracing data have suggested, however, that the epithelium may alternatively be maintained by committed progenitor cells and that individual cell fate is determined at random (21, 22). It has been suggested that PV could modulate the infected cell, giving it stem-like properties, such as upregulation of stem cell marker genes or delayed differentiation as a result of the inhibition of the Notch signaling pathway (23–25). In such ways, keratinocytes infected with PV may develop stem-like traits following an infection. Understanding this element of basal cell homeostasis during early papillomavirus lesion formation and persistence appears to be important in the development of therapeutics targeting the persistently infected basal cell.

Although unclear, it is plausible that only a single infected cell is enough to establish a new lesion. Therefore, understanding what competitive advantages such a cell must have in order to develop a lesion and how it can persist in the basal layer is important; ascertaining the way in which single infected cells compete with uninfected neighbors to form lesions could reveal key mechanisms of infection. These mechanisms may also explain how certain HPV types can establish productive lesions without the pro-proliferative capabilities of their high-risk counterparts. Both *in vivo* and *in vitro*, Notch signaling has been shown to act as a key determinant in the coordination of keratinocyte transition from proliferation to early-stage differentiation phenotypes (26). Inactivation studies have demonstrated the role of Notch signaling in regulation of late-stage differentiation in keratinocytes (27). Other studies have also shown the involvement of Notch signaling in the differentiation process (26, 28). Notch signaling plays a vital role in successful maintenance of keratinocytes in normal epithelium; given the previously discussed ability of PVs to interact with this pathway, it is clear that Notch pathway interaction could be implicated in PV modulation of the cell to successfully persist in hosts.

Here, we study the characteristics of infected cells with a view to understand how infections develop and persist, by examining and modeling the generic characteristics of PV infection using the mouse model of papillomavirus infection. Given the broader life cycle strategies shared by all PV types, MmuPV1 provides a useful model with which to study aspects of persistence and cell competition *in vivo* by examining the mechanics of basal cell expansion. We have identified four key stages during papillomavirus lesion formation. A delay in differentiation and an increase in cell density are early viral modifications during this process. As lesions become established, a weak bimodality in E6/E7 expression becomes apparent in infected basal cells, with higher expression levels correlating with delamination, migration into the parabasal cell layers in the absence of accompanying differentiation, and the induction of K10 expression. Our results suggest that E6 is responsible for these phenotypic changes, with the protein conferring a preferential advantage over neighboring uninfected cells, which, in *in vitro* assays, are displaced into the stratified cell layers. This competitive advantage, which is mediated in part through modulation of the Notch pathway, points to an important role for E6 in establishing and maintain the reservoir of papillomavirus infection in the epithelial basal layer.

## RESULTS

### Stages of papillomavirus lesion formation in immunodeficient and immunocompetent mice.

**FIG 1:**
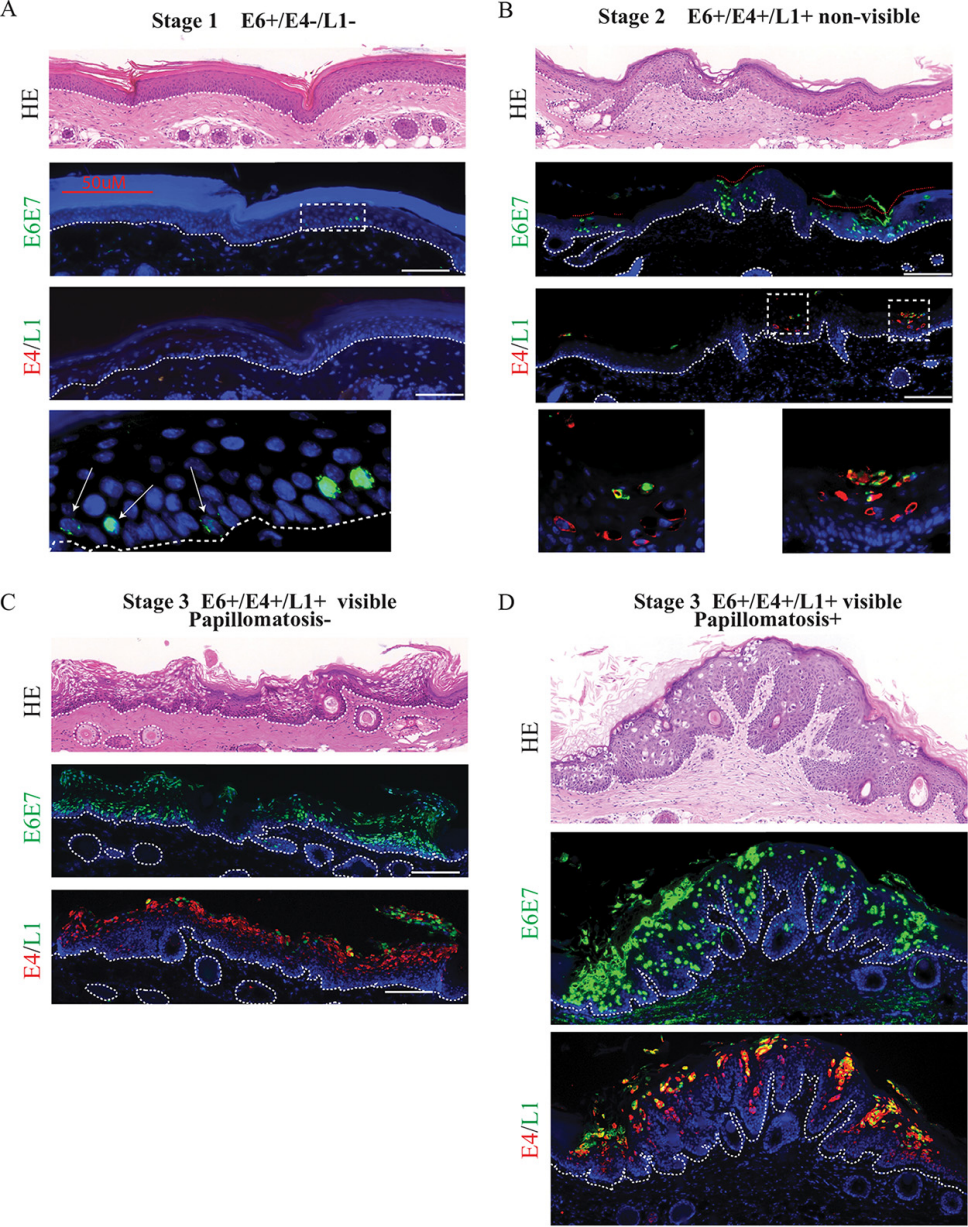
Spatial localization of MmuPV1 E6/E7 expression and E4 and L1 proteins in early lesion formation of infected tissues. (A to D) Time course analysis of early lesion formation of sites infected with MmuPV1 cell-free virus. Each set of panels shows hematoxylin and eosin (HE) staining (top), E6/E7 RNAScope signal (middle), and immunofluorescent detection of E4 and L1 proteins (bottom) at a discrete stage of lesion formation. Staining was carried out on adjacent sections. The nuclei were counterstained with DAPI. The scale is shown with a white bar (100 μm). The boxed areas are enlarged at the bottom (A and B). The dotted lines indicate the position of the basal layer.

Our previous report showed that early visible lesion formation can be observed at day 6 or 7 postinoculation of high-titer viruses (29). To observe the process of lesion formation, nude mice were scarified at three discrete regions along the tail, and approximately 2 × 10^9^ VGE MmuPV1 cell-free virus was introduced at each site. To identify the earliest stages of lesion formation, mouse tails were collected between days 1 and 5 postinoculation. When lesions became macroscopically visible (early visible) at any site on day 6 or 7 postinoculation, the entire tail was collected, allowing the identification of “pre-visible lesions” present elsewhere on the same tail. The well-established lesions (warts) were also collected 10 to 14 days postinoculation. In Fig. 1, four discrete stages of lesion formation are shown.

Stage 1 depicts the earliest detectable lesion events (Fig. 1A). Of the genes examined, only expression of E6/E7 could be detected, which was restricted to a subset of basal and parabasal cells and was apparent as early as day 2 postinoculation. Among the 48 samples collected, E6/E7 expression was only seen in regions where reepithelialization was complete, suggesting that the early stages of wound healing must be completed before viral gene expression begins. In stage 2 (Fig. 1B), the first evidence of MmuPV1 virus production is apparent in the uppermost layers, shown by the expression of viral capsid protein, suggesting the development of productive lesions without macroscopic/visible change. Additional discrete foci of infection (red dotted lines) became apparent as lesion development progressed, suggesting that larger lesions develop from multiple discrete foci, which coalesce to form one lesion. In stage 3 (Fig. 1C), the lesion becomes macroscopically visible for the first time. We observed high basal cell density at the lesion site, as seen throughout the basal layer of the example shown, and referred to here as “early visible lesions.” In this example, one continuous region of infection has developed, apparently from multiple foci visible at the previous stage. Finally, in stage 4 of lesion formation, we see induction of papillomatosis and warts macroscopically, subsequently referred to here as “established lesions.”

**FIG 2:**
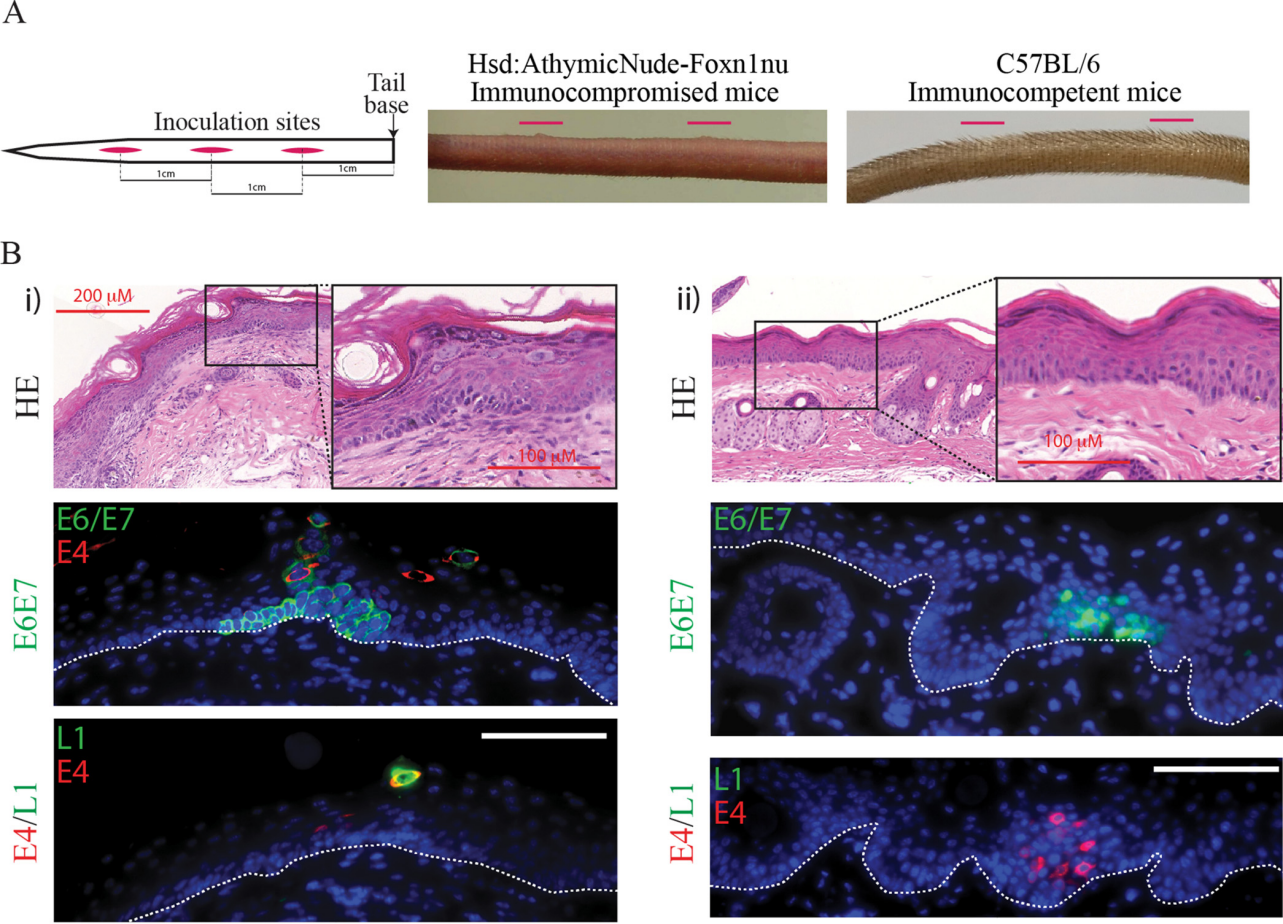
Identification of productive MmuPV1 lesions in immunocompetent mice. (A) Diagrammatic representation of inoculation site procedure in our model (left panel). Inoculation sites are shown in red, and the 1-cm spaces between the center of each wound site are annotated. The first wound site is located 1 cm down from the base of the tail. There is early visible lesion formation on immunodeficient nude mouse tail 10 days postinfection: sites are indicated with red lines (center panel). C57BL/6 immunocompetent mice showed no evidence of papilloma formation at wound sites 10 days postinfection (right panel). (B) Transient lesions located in C57BL/6 immunocompetent mouse tail tissue wound site 7 and 10 days following inoculation (i and ii). Panels show HE staining (top panel), E6/E7 RNAScope immunofluorescence and E4 protein (center), and immunofluorescent detection of E4 and L1 proteins (bottom panel). The nuclei were counterstained with DAPI. The scale is shown with a white bar (100 μm). The boxed areas in HE staining are enlarged. The dotted lines indicate the position of the basal layer.

Infection of C57BL/6 immunocompetent mice with MmuPV1 inoculation did not result in the formation of papillomas at the tail sites, which is in contrast to the macroscopically visible lesions that form in nude mice (Fig. 2A). A thorough analysis of tissue samples led to the identification of productive microlesions in the C57BL/6 mouse tail tissue at the site of infection at 10 days postinoculation (Fig. 2B). As shown in Fig. 2Bi, expression of E6/E7 RNA was evident in the epithelial basal cells and persisted into the upper layers of the epidermis toward the skin surface. This prominent cytoplasmic distribution of virus transcripts differs from the less clearly defined basal expression seen in our athymic nude immunodeficient model, which was predominantly nuclear. Similarly, the E4 protein can be seen colocalizing to this area, confirming the presence of a PV-induced microlesion. Finally, immunofluorescent staining identified the presence of occasional L1-expressing cells in the upper layers, which suggests the development of a productive lesion (Fig. 2Bi). Figure 2Bii shows a second example of a lesion located at a wound site 7 days postwounding. While E6/E7 RNA and E4 protein are also both present at this site, L1 could not be located. Therefore, these lesions in the immunocompetent animals are understood to have reached stage 2 of lesion formation.

### MmuPV1 modulation of postinfection basal cell density.

**FIG 3:**
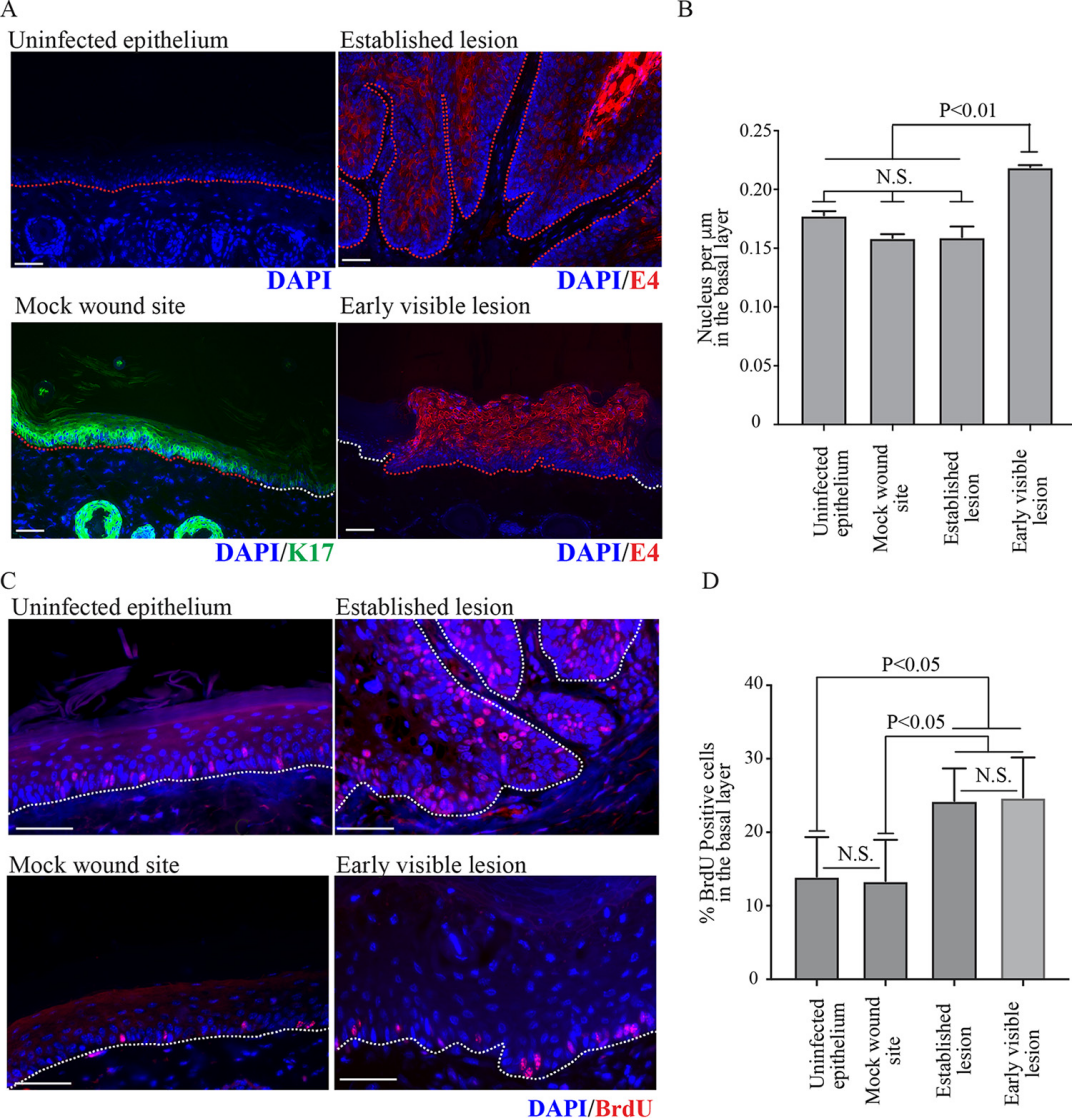
Differences in basal cell density and DNA replication in developing lesions. (A) Representative images of the four categories uninfected epithelium, mock wound sites, early visible lesions in nude mouse tissue (samples collected 6 or 7 days postinfection, validated using E6/E7 RNAScope), and established lesions in tissue (samples collected from tail lesions more than 10 days postinfection) are shown. Infected lesion was identified by E4 expression, and the wounded area was identified by K17 expression. The nuclei were counterstained with DAPI. The white dotted lines indicate location of basement membranes, and the red dotted line indicates the areas that were measured. The scale is shown with a white bar (200 μm). (B) Basal cell density was quantified by measuring 450-μm stretches of epithelium in 40 image sets taken from different nude mice (*n* = 4 per category). *P* values were calculated with Kruskal-Wallis test with Dunn’s correction. (C) Representative images are shown (right panel) for each category. Proliferating cells were visualized by BrdU staining. The nuclei were counterstained with DAPI. White dotted lines indicate the location of basement membranes. The scale is shown with a white bar (50 μm). (D) The percentage of BrdU-positive cells in the basal layer of the above four categories (*n* = 3). *P* values were calculated with Kruskal-Wallis test with Dunn’s correction.

An apparent correlation between the appearance of E6/E7 RNA expression and increase in basal cell density was observed from stage 2 of lesion formation onwards. Since this phenotype was seen early during lesion formation in the basal layer, involvement of a viral early protein was suspected. To examine this further, a more quantitative analysis was carried out, with uninfected and mock-infected epithelium serving as controls. Two time points during lesion formation were examined in order to establish basic principles: “early visible lesions,” which are equivalent to stage 3 during lesion formation (Fig. 1C), and “established lesions,” which are florid tail lesions collected more than 10 days postinfection (Fig. 1D). For the early visible lesion analysis, macroscopically visible lesions that were collected 6 or 7 days following virus inoculation and which were shown to express viral E4 proteins (or E6/E7 RNA analysis) were used (Fig. 3A). Time-matched mock-infected sites, which were shown to express reepithelialization keratin 17 (Fig. 3A) (30), were selected for comparison with the early visible lesions. For quantification, 40 image sets of 4′,6-diamidino-2-phenylindole (DAPI)-stained tissue were collected for each category (i.e., uninfected epithelium, mock wound site, early visible lesions, and established lesions [mice, *n* = 4 per group]), and basal cell number was counted in 450-μm stretches in each image (red lines in Fig. 3A). Basal cell density was found to be elevated in early visible lesions compared to all other categories, and averaged 0.22 cell per μm (*P* ≤ 0.01) (Fig. 3B). In contrast, uninfected epithelium and mock wound sites averaged 0.18 and 0.16 basal cells per μm, respectively, with basal cell density at uninfected epithelial sites being similar to that observed in established lesions (0.16 cells per μm).

To investigate this phenomenon in more detail, we proceeded to establish if the number of cells driven to replicate their DNA was altered in the virus-infected basal cells, and whether this differed between early visible and established lesions. Bromodeoxyuridine (BrdU) incorporation was monitored by collecting tissue samples 24 h after intraperitoneal injection of BrdU by immunofluorescence (Fig. 3C), and the proportion of BrdU-positive basal cells in uninfected epithelium, mock wound, early visible lesions, and established lesions was established. A statistically significant increase in the proportion of BrdU-positive cells in the basal layer in the stage 5/early visible lesion was apparent compared to uninfected epithelium (Fig. 3D). In both uninfected tail epithelium and mock-infected tail sites, approximately 14% and 13% of basal cells were BrdU positive, respectively, with no significant difference apparent between the two groups. In the early visible lesions, 25% of cells in the basal layer were positive for BrdU following our labeling regime. There was no significant difference in the percentage of BrdU-positive basal cells between early visible lesions and established lesions, in which 24% of basal cells were positive for BrdU. The percentage of BrdU-positive cells in early visible and established lesions was significantly higher than in both uninfected epithelium and mock-wounded epithelium (*P* < 0.01). Although the percentages of BrdU-positive cells may be expected to differ between mock-infected and uninfected epithelium, reepithelialization has been largely completed at this point, and the percentages of replication-competent cells that become labeled during the 24-h period were similar. These results suggest that the elevated basal cell density observed in early visible lesions is unlikely to be a consequence of inherent differences in cell cycle progression and proliferation, prompting us to look at viral gene expression in relation to basal cell retention and differentiation.

### E6/E7 expression correlates with a delay in normal differentiation in the parabasal layers of infected epithelium.

**FIG 4:**
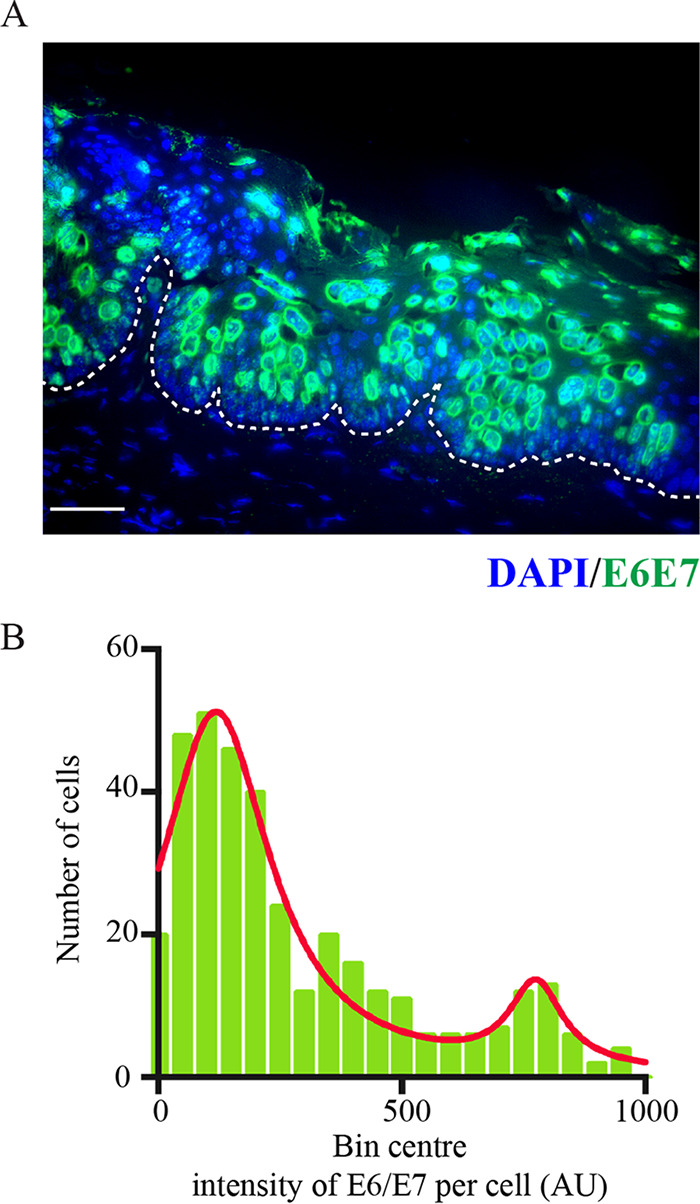
Heterogeneous expression pattern of E6/E7 RNA in the basal layer. (A) Representative images of viral E6/E7 RNAScope in the early visible lesion. The dotted lines indicate location of basement membranes. The scale is shown with a white bar (50 μm). (B) Distribution of viral RNA expression levels in basal cells of early visible lesions (*n* = 4) is shown in the histogram. Intensity of expression per cell (AU) is grouped into bin centers for bimodal analysis. A sum of 2 Lorentzian distribution lines is fit to the data (red).

As the above analysis suggests that an increase in basal cell density could not be attributed to increased cell replication alone, it was postulated that MmuPV1 E6/E7 expression may lead to persistence of infected cells in the basal layer by overcoming normal cell density modulation and contact inhibition. To investigate this in our time course of lesion formation, the expression level of E6/E7 RNA in individual cells was evaluated in the basal cell layers across stage 3, early visible lesions. Following quantification of the level of E6/E7 RNA (Fig. 4A and B), a weak bimodality was apparent in E6/E7 RNA expression levels of the basal cells, suggesting the presence of two discrete cell populations, with the majority expressing these viral genes at a low level. Although a test of bimodality did not confirm statistical significance (Hartigan’s dip test, *P* = 0.914), the data were significantly not normal when all three normality tests were applied (D’Agostino and Pearson normality test, *P* ≤ 0.0001; Shapiro Wilk normality test, *P* ≤ 0.0001; KS normality test, *P* ≤ 0.0001). The sum of two Lorentzian curves (shown in red on the histogram in Fig. 4B) closely fitted our observational data.

**FIG 5:**
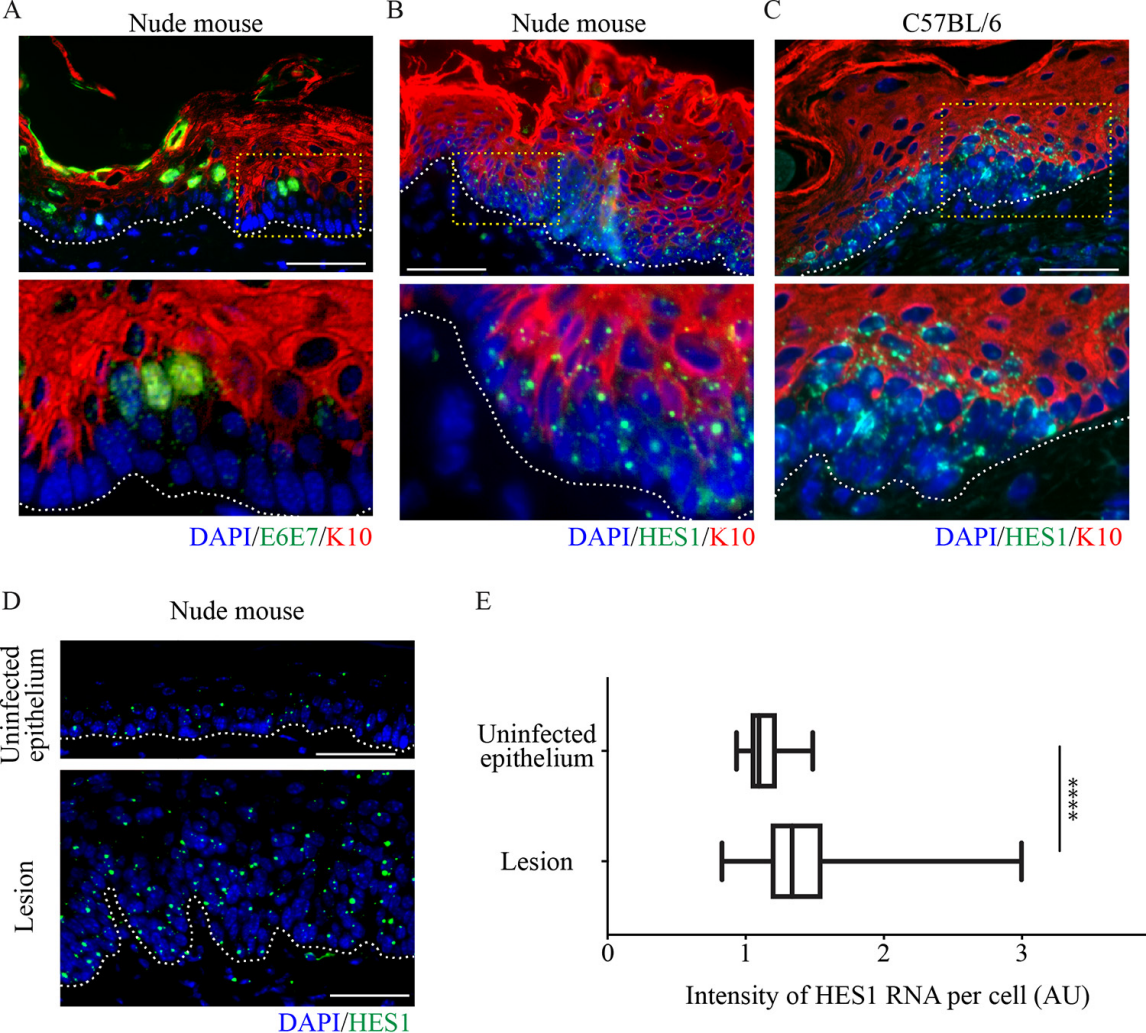
Delay in normal differentiation induction occurs in tissue in E6/E7 RNA-positive cells (A and B) An early visible lesion from a nude mouse was stained for K10 (red), E6/E7 RNAScope (A), or HES-1 RNAScope (B) (green) and nuclei with DAPI (blue). The dotted lines indicate the position of the basal layer. The yellow boxed area is enlarged at the bottom. Scale bar, 100 μm. (C) A microlesion from a C57BL/6 mouse (Fig. 2Bi) stained for K10 (red), HES-1 RNAScope (green), and nuclei with DAPI (blue). The dotted lines indicate the position of the basal layer. The yellow boxed area is enlarged at the bottom. Scale bar, 100 μm. (D) An uninfected epithelium (top) and an infected lesion (bottom) were stained for HES-1 RNAScope (green) and DAPI (blue). The dotted lines indicate the position of the basal layer. Scale bar, 100 μm. (E) Box plot to show intensity of HES-1 RNA expression in the basal layer of cells of uninfected epithelium (*n* = 10) and lesion (*n* = 10). *P* values were calculated with a Kolmogorov-Smirnov *t* test. ****, *P* ≤ 0.0001.

To examine the involvement of E6/E7 RNA expression in basal cell exit and differentiation, two markers of epithelial differentiation were used; K10, an established early-stage marker of cells entering terminal differentiation, and HES1, a downstream target of the Notch signaling pathway (28), which is also known to be a target of MmuPV E6 (25). Double staining for MmuPV1 E6/E7 RNA and K10 demonstrated that in the presence of the early MmuPV1 E6/E7 RNA, K10 is completely absent (Fig. 5A) in the second layer. Expression of HES1 RNA was not noticeably delayed in these areas of delayed differentiation compared to surrounding normal epithelium, and was expressed throughout the basal and parabasal layers (Fig. 5B). As seen in the experiments carried out with immunodeficient mice, there is also a decrease in K10 staining that correlates with the elevated HES1 phenotype present in immunocompetent microlesions (Fig. 5C). In fact, quantification of HES1 RNA expression per cell in the basal layer of lesions versus uninfected tissue demonstrated a significantly higher level of HES1 RNA expression in the infected basal cells (Fig. 5D and E), suggesting that while differentiation was retarded, the Notch signaling pathway was active. Therefore, it is not total inhibition of the Notch pathway by E6/E7 that leads to the observed delay in differentiation, where cells exited the basal layer without differentiating. These data indicated that in both immunocompetent and immunosuppressed backgrounds, MmuPV1 infection delayed differentiation commitment in the basal and parabasal layers, which we suspect will enhance basal layer persistence of the infected cell. E6 has previously been speculated to regulate commitment to differentiation (24), therefore it was decided that examination of this phenotype in a cell culture model wherein individual protein functions can be discretely analyzed would be carried out.

### Keratinocytes expressing MmuPV1 E6, but not E7, show higher saturation density in 2D culture.

To overcome some of the complexities encountered in *in vivo* experiments, cell culture systems were introduced to add clarity and to further explore our emerging hypotheses. To determine which of the viral proteins confers the *in vivo* cell density and differentiation phenotypes observed, normal immortalized keratinocyte (NIKS) cell lines exogenously expressing MmuPV1 E6 (NIKS/LXSN-MmuPV1E6) or MmuPV1 E7 (NIKS/LXSN-MmuPV1E7) were generated. NIKS cells containing an empty LXSN vector were used as a control (NIKS/LXSN).

**FIG 6:**
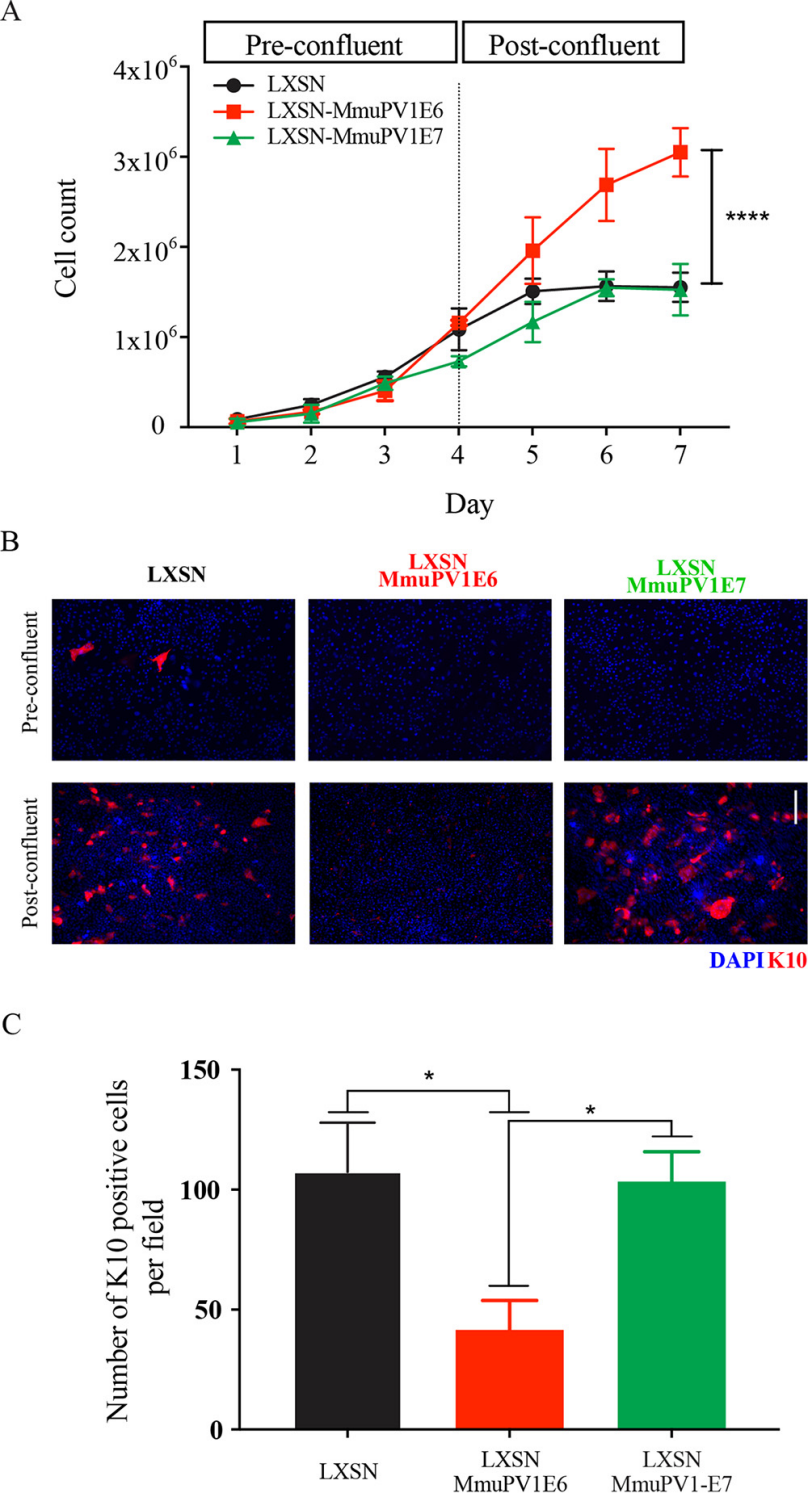
Exogenous expression of MmuPV1 E6 leads to higher cell density and differentiation delay in keratinocytes. (A) NIKS/LXSN or NIKS cells expressing MmuPV1 E6 or E7 were counted in triplicate every day. Cells were confluent around day 4. *P* values were calculated with a two-way analysis of variance (ANOVA) with Tukey’s correction. ****, *P* ≤ 0.0001. (B) Cells were stained with K10 (red) and DAPI (blue) at both preconfluence (day 3) and postconfluence (day 7). Scale bar, 100 μm. Light microscope images for each time point are also shown. Red arrows denote the presence of ring-like structures in cell monolayer morphology. Black arrows denote the presence of bright, rounded cells. (C) The number of K10-positive cells per field for each cell population. *P* values were calculated with Kruskal-Wallis test with Dunn’s correction. *, *P* ≤ 0.05.

As an increase in cell density and cell proliferation had been quantified *in vivo*, two-dimensional (2D) monolayer growth assays were carried out to establish the role of each protein *in vitro* (Fig. 6A). From days 1 to 3, before cells had reached confluence, there was no significant difference in the growth rates of cells across all three groups. After cells had reached confluence (days 4 to 6), the increase in cell counts of NIKS/LXSN and NIKS/LXSN-MmPV1E7 plateaued, and at day 7, there was no significant difference—1.55 × 10^6^ and 1.53 × 10^6^ cells, respectively. In contrast, NIKS/LXSN-MmuPV1E6 cells reached 3.1 × 10^6^, a significantly higher density compared to the other two groups (****, *P* ≤ 0.0001), demonstrating that MmuPV1 E6 plays an important role in maintaining the proliferative capacity of the cell as cell density increases.

As shown in Fig. 6B, the morphological appearances of the NIKS/LXSN and NIKS/LXSN-MmuPV1E7 cells by day 7 were highly differentiated (shown with red arrows), whereas in NIKS/LXSN-MmuPV1E6 cells, the small, bright, rounded cells are indicative of cells still undergoing replication (denoted with black arrows). Analysis of K10 staining in all three populations indicated that at low densities almost all cells lacked K10. However, at high density, while NIKS/LXSN and NIKS/LXSN-MmuPV1E7 cells showed a similar level of K10 expression per field, NIKS/MmuPV1E6-LXSN cells had noticeably lower expression levels of K10. Quantification analysis showed that when data were normalized by the total number of cells per field to calculate the percentage of positive cells per field, there was a statistically significant decrease in the percentage of K10-positive cells per field (**, *P* ≤ 0.01) in NIKS/MmuPV1E6-LXSN cells compared to the other two groups (Fig. 6C). This shows that the effect of MmuPV1 on differentiation seen *in vivo* is also seen in the cell culture model and that MmuPV1 E6 is responsible for this phenotypic change.

### Keratinocytes expressing MmuPV1 E6, but not E7 persist in the “lower” layer of cells in a high cell density culture environment.

**FIG 7:**
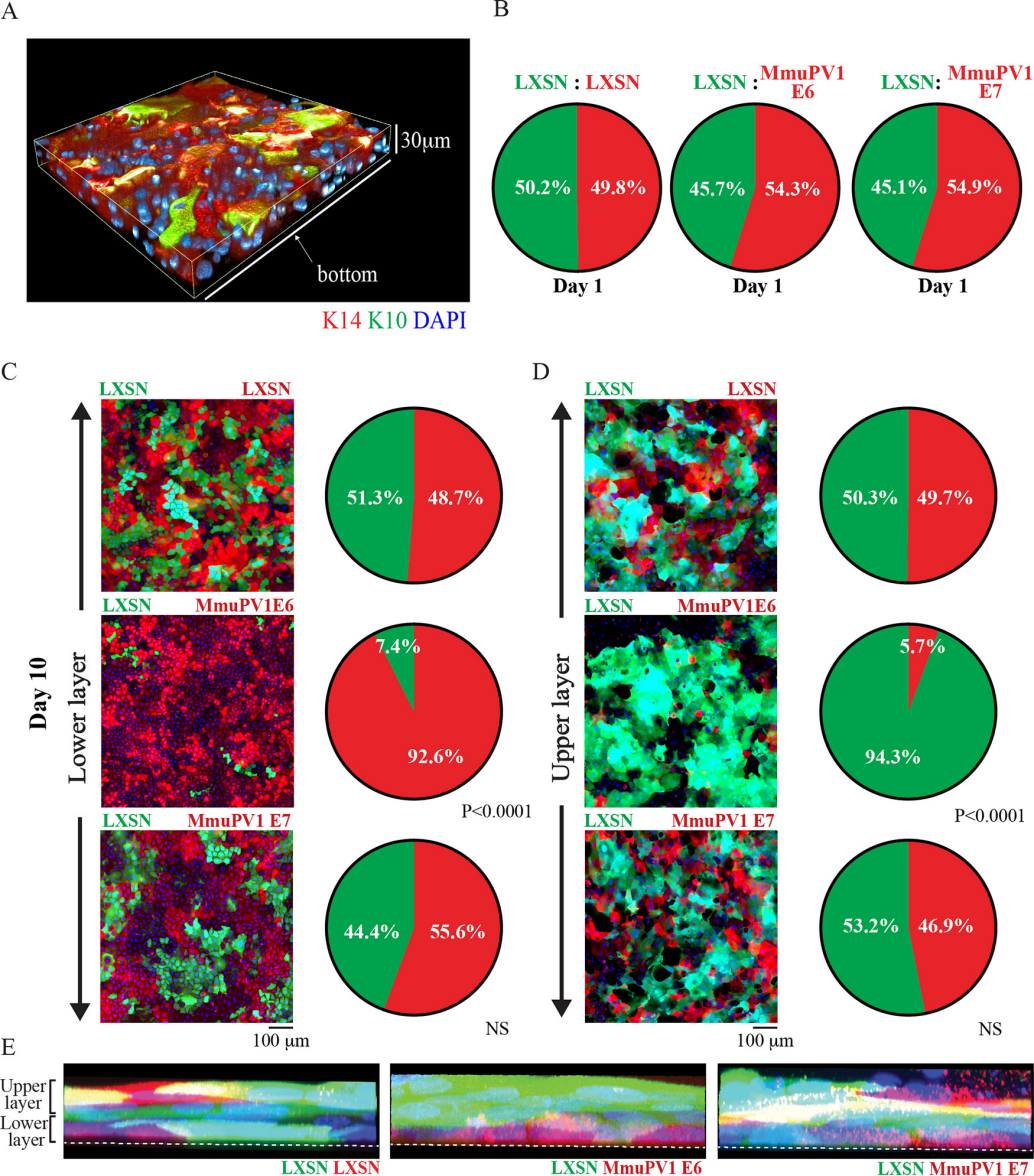
NIKS cells expressing MmuPV1 E6 preferentially persist in the lower layer of cells in a high-density competition assay. (A) NIKS cells were seeded at high density and cultured for 7 days. A 3D image of NIKS cells at day 7 stained with K14 and K10 is shown. (B) The same number of cells of each cell line was seeded together at day 0, and the proportions of each cell line of LXSN (green) to LXSN (red), LXSN (green) to LXSN-MmuPV1E6 (red), and LXSN (green) to LXSN-MmuPV1E7 (red) at day 1 are shown. (C and D) Representative images of the lower layer (C) and upper layer (D) of each group are shown (left), alongside the proportions of each cell line of each group (right) at day 10. Experimental groups were compared with LXSN/LXSN control groups, and *P* values were calculated with a two-way ANOVA with Sidak's multiple-comparison test. NS, not significant. Scale bar, 100 μm. (E) Maximum-intensity 3D plots of z-stacks are shown for each of the three groups. Annotations show the location of the lower and upper layers. White dotted lines indicate the bottom of the slide glass on which the cells are cultured. The nuclei were counterstained with DAPI.

**FIG 8:**
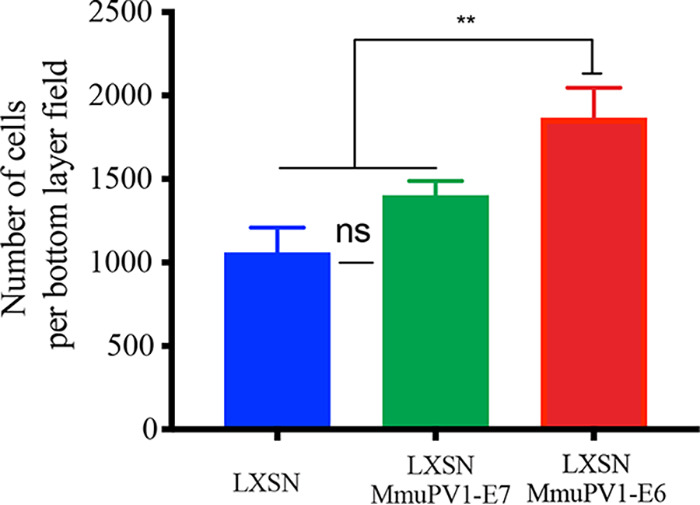
Viral protein expression can alter cell density of the “lower” layer of cells. Quantification of the number of cells in the bottom layer of each population (field size, 800.22 by 800.22 mm). Five random 5-by-5 tile scans were chosen for quantification for each group at the day 7 time point. *P* values were calculated with Kolmogorov-Smirnov *t* tests. **, *P* ≤ 0.01; ns, not significant.

Thus far, our work implicates MmuPV1 E6 in the differentiation delay and increased cell density observed in our *in vivo* model. As our results suggested that increased cell proliferation was not responsible for the observed increase in cell density (Fig. 3), we wanted to investigate the idea that preferential persistence of infected cells in the basal layer could lead to the increased density observed as lesions first begin to form. To investigate this, we established NIKS/LXSN, NIKS/LXSN-MmuPV1E6-LXSN, and NIKS/LXSN-MmuPV1E7 cells expressing enhanced green fluorescent protein (eGFP) or mCherry to observe their relative cell growth characteristics over time by culturing them together to produce a cell competition assay system. In this assay, at the start the two cell types were seeded at the same high density to overcome the difference in expansion growth characteristics of each cell line, so that the different cell populations grew from confluence onwards. Under this culture condition, NIKS cells start to express K10 in second-layer cells only but K14 in both layers (Fig. 7A). The expression pattern is observed in organotypic raft culture tissue of NIKS (24). This competition assay system provides a crude model of a confluent basal layer; cells in this culture are confluent, with no space to grow, which is the same spatial environment in which basal cells naturally exist. For each well, half of the population seeded were NIKS/QCXIP-eGFP/LXSN cells, the other half being NIKS/QCXIP-mCherry/LXSN, NIKS/QCXIP-mCherry/LXSN-MmuPV1E6, or NIKS/QCXIP-mCherry/LXSN-MmuPV1E7. Cells were cultured for up to 10 days, with the growth of these mixed-cell populations observed by confocal microscopy. At day 1 (Fig. 7B), all three experimental groups had an approximate 50:50 ratio of red versus green cells, as expected. A slight increase in red cells in both the NIKS/QCXIP-eGFP/LXSN cells versus NIKS/QCXIP-mCherry/LXSN-MmuPV1E6 cells (LXSN/LXSN-MmuPV1E6) group and the NIKS/QCXIP-eGFP/LXSN cells versus NIKS/QCXIP-mCherry/LXSN-MmuPV1E7 (LXSN/LXSN-MmuPV1E7) group was apparent, and although not statistically significant, suggests a slight advantage in cell attachment or cell growth over control cells in the first 24 h. At day 10 (Fig. 7C and D), there were approximately 50% of each cell population occupying both the lower and upper layers of cells in the NIKS/QCXP-eGFP/LXSN versus NIKS/QCXIP-mCherry/LXSN (LXSN/LXSN) group and in the LXSN/LXSN-MmuPV1E7 group. Conversely, within the LXSN/LXSN-MmuPV1E6 group, the vast majority of cells occupying the lower layer of cells in culture were NIKS/QCXIP-mCherry/LXSN-MmuPV1E6 cells (92.6% red cells), while the upper layer of cells consisted almost entirely of NIKS/QCXIP-eGFP/LXSN cells (94.3% green cells). This suggested that NIKS expressing MmuPV1E6 were preferentially persisting in the lower layer of cells. To observe these phenotypes in 3D, maximum-intensity side views of orthologue plots for each cell population are shown (Fig. 7E). This again demonstrates that in the LXSN/LXSN and LXSN/LXSN-MmuPV1E7 groups, each cell type can be seen distributed in roughly equal measure in both layers. Conversely, in the LXSN/LXSN-MmuPV1E6 group, a lower layer of MmuPV1E6-expressing cells can clearly be seen, while the upper layer consists of almost exclusively LXSN control cells. Taken together, these data show that expression of MmuPV1 E6, but not E7, affords the cell a growth advantage or a capability of remaining at the bottom layer over the control cell population when in direct competition for space. Actually, quantitative analysis of the lower layer of cells in each of these groups was carried out at day 10 (Fig. 8). Data demonstrated that the NIKS/LXSN-MmuPV1E6 group reached a significantly higher density in the lower layer of cells compared to the separately grown NIKS/LXSN and LXSN/LXSNMmuPV1E7. These data corroborate earlier results both *in vivo* and *in vitro* demonstrating that MmuPV1 E6-expressing cells can grow to higher cell densities than control cells, and also suggest that increased basal cell density observed in the early mouse lesions (Fig. 3A and B) is due primarily to MmuPV1 E6 expression. Finally, the data could suggest that, in addition to the cell density, the rate at which cells are exiting this bottom layer may be affected.

### MAML binding-deficient MmuPV1 E6 loses ability to persist in the “lower” layer.

**FIG 9:**
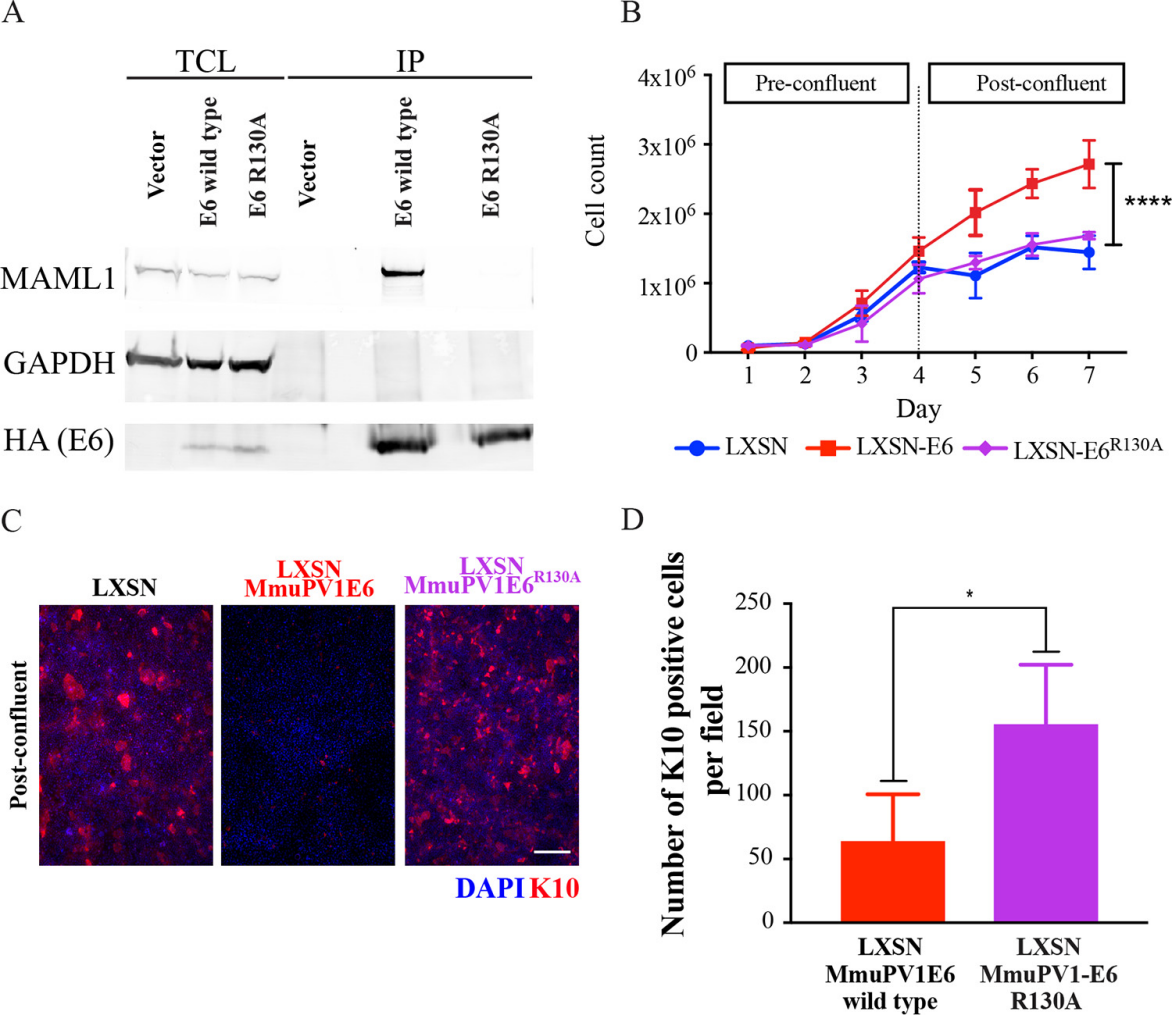
MmuPV1 E6 interaction with MAML1 is required for postconfluent density increase and differentiation delay. (A) Lysates of 293TT cells expressing HA-tagged wild-type or mutant MmuPV1 E6 protein were subjected to an immunoprecipitation using HA antibody. Immunoprecipitated MmuPV1 E6 and associated MAML1 were detected by immunoblotting. Expression levels of proteins were assessed by immunoblotting of total cell lysate (TCL). GAPDH expression was used as a loading control. (B) NIKS/LXSN, NIKS/LXSN-MmuPV1E6, or NIKS/LXSN-MmuPV1E6^R130A^ cells were counted each day. Cells were confluent around day 4. *P* values were calculated with a two-way ANOVA with Tukey’s correction. ****, *P* ≤ 0.0001. (C) NIKS/LXSN, NIKS/LXSN-MmuPV1E6, or NIKS/LXSN-MmuPV1E6^R130A^ cells were stained with K10 (red) at both preconfluence (day 3) and postconfluence (day 7). Nuclei were counterstained with DAPI. Scale bar, 100 μm. Light microscope images for each time point are also shown for all three cell populations. Red arrows denote presence of ring-like structures in cell monolayer morphology. (D) The number of K10-positive cells per field for NIKS/LXSN-MmuPV1E6 or NIKS/LXSN-MmuPV1E6^R130A^ cells. *P* values were calculated with a Kruskal-Wallis test with Dunn’s correction. *, *P* ≤ 0.05.

To further understand the molecular pathways involved in directing this particular competitive advantage of MmuPV1 E6-expressing cells, a mutant MmuPV1 E6 was generated. Previously published research into the similarities between MmuPV1 E6 and HPV8 E6 interactions with the Notch pathway confirmed that MmuPV1 E6 was able to bind to MAML, and that this interaction delayed differentiation in Ca^2+^-treated keratinocytes (25). It was shown that an E6 MAML binding mutant could not inhibit Notch signaling and that this mutant was unable to form papillomas *in vivo.* As such, it was postulated that this pathway may be involved in the phenotype observed in the high-density competition assay, and that similar interference with MmuPV1 E6 MAML1 binding in this model could indicate whether the downstream Notch signaling pathway was involved in modulation of the persistence phenotype. Therefore, a MAML1 binding mutant of MmuPV1 E6, MmuPV1^R130A^, was generated. Using an immunoprecipitation assay, it was confirmed that the MmuPV1-E6^R130A^ mutant had a reduced ability to bind to MAML1 (Fig. 9A). NIKS cells expressing MmuPV1-E6^R130A^ had no significant difference in cell density at day 7 compared to LXSN-expressing cells, unlike MmuPV1 E6-expressing cells, which grew to a significantly higher density (****, *P* ≤ 0.0001) by this time point (Fig. 9B). Furthermore, cells expressing MmuPV1-E6^R130A^ no longer showed a delay in differentiation postconfluence when stained with K10 (Fig. 9C). Quantification of the number of K10-positive cells (Fig. 9D) demonstrated that the percentage of K10-positive cells per field was significantly lower in the population of NIKS/MmuPV1E6^R130A^-LXSN cells (**, *P* ≤ 0.05) compared to the other groups, suggesting that the delay in differentiation observed and quantified in NIKS/LXSN-MmuPV1E6 cells is lost upon introduction of the E6^R130A^ MAML1 binding mutation.

**FIG 10:**
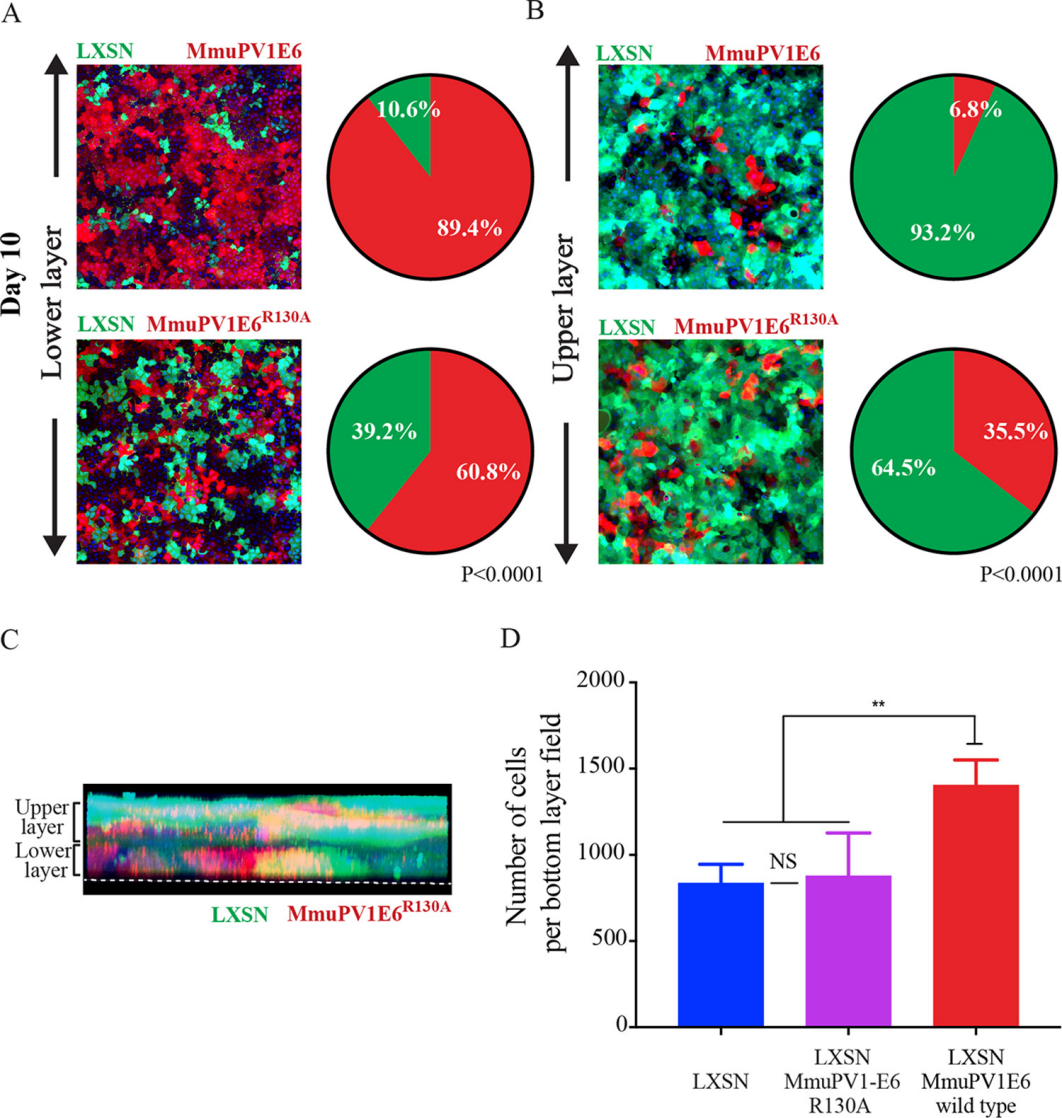
MmuPV1 E6 interaction with MAML1 is required for preferential persistence in the lower layer of cells in the high-density competition assay. (A and B) Representative images of the lower layer (A) and upper layer (B) of each group are shown (left), alongside the proportions of each cell lines of each group (right). Experimental groups were compared each other, and *P* values were calculated with a two-way ANOVA with Sidak's multiple-comparison test. NS, not significant. Scale bar, 100 μm. (C) Maximum-intensity 3D plots of z-stacks are shown for each group. Annotations show the location of the lower and upper layers. White dotted lines indicate the bottom of the slide glass on which the cells are cultured. The nuclei were counterstained with DAPI. (D) Quantification of the number of cells in the bottom layer of each population (field size, 800.22 by 800.22 μm). Five random 5-by-5 tile scans were chosen for quantification for each group at a day 7 time point. *P* values were calculated with Kolmogorov-Smirnov *t* tests. **, *P* ≤ 0.01; NS, not significant.

Again, the cell competition assay and layer analysis were repeated with NIKS/QCXIP-mCherry/LXSN-MmuPV1E6^R130A^ cells versus NIKS/QCXIP-eGFP/LXSN cells (LXSN/LXSN-MmuPV1E6^R130A^). Results of the cell competition assay are shown in Fig. 10A and B. In the LXSN/LXSN-MmuPV1E6^R130A^ group, there was a statistically significant difference (****, *P* ≤ 0.0001) between layer occupancy compared to LXSN/LXSN-MmuPV1E6 groups at day 10. While the lower-layer occupancy was 89.4% NIKS/QCXIP-mCherry/LXSN-MmuPV1E6 cells, this was reduced to 60.8% for the MAML1 NIKS/QCXIP-mCherry/MmuPV1E6^R130A^ cells. Similarly, the upper layer of the LXSN/LXSN-MmuPV1E6 group consisted of 93.2% eGFP-positive (NIKS/PQCXIP-eGFP/LXSN) cells, whereas this decreased to only 64.5% in the LXSN/LXSN-MmuPV1E6^R130A^ group. This phenotype is also confirmed in 3D (z-stack image) (Fig. 10C). The NIKS/LXSN-MmuPV1E6^R130A^ mutant cell line did not retain the ability of wild-type E6 to persist preferentially in the lower layer, instead demonstrating a random assortment of eGFP-positive and mCherry-positive cells in the lower and upper layers (Fig. 10C). Finally, quantification of cell density in the lower layer of the LXSN/LXSN-MmuPV1E6^R130A^ group showed that the increase in lower-layer density previously observed in the LXSN/LXSN-MmuPV1E6 group (Fig. 8) was also lost following the disruption of MmuPV1 E6 MAML1 binding, as there was no significant difference between the LXSN/LXSN-MmuPV1 E6^R130A^ and NIKS/LXSN groups in lower layer cell density at day 10 (Fig. 8 and Fig. 10D). These data clearly demonstrated that MmuPV1 E6 interference with the Notch pathway via interaction with MAML1 is necessary to allow cells to preferentially persist in the lower layer of cells and may also mediate the effect of MmuPV1E6 on basal lower cell layer density regulation.

## DISCUSSION

Papillomaviruses exclusively infect the stratified squamous epithelium to induce chronic infection in their host (31). Despite advancements in the field, the earliest events in lesion formation (infection of a cell, initial stages of lesion development, and the associated molecular mechanisms), are less well understood. This article aims to investigate the initial events in lesion formation to better understand how, in these first stages of infection, single infected cells can persist and outcompete uninfected cells.

The incubation period of papillomaviruses from exposure to lesion development varies greatly, the usual range being between 1 and 20 months (32). Initial events during the incubation period are still unknown, and it has been proposed that co-cofactors (e.g., host/local immunity, synergistic infection, inflammation or tobacco use) may be required for lesion initiation (33), and if the cofactor is not present, the papillomavirus may lie in dormant/latent infection and only express itself after the cofactor appears. In our previous study using the papillomavirus mouse model (29), however, the incubation period in nude mice was basically determined by the virus titer inoculated. Lesions formed in 1 week in mice inoculated with high virus titer, whereas with low-titer lesion formation took 12 weeks. This suggested that the number of initial infection events may define how quickly the lesion forms (becomes apparent). Indeed, we observed that when high-titer virus was inoculated, lesions initially started from multiple microfoci of infected cells surrounded by noninfected cells. These microfoci expanded in the basal layer to form one continuous region of infected cells before becoming apparent (Fig. 1B). This observation supports the idea that PV infection and expression of virus genes confer “fitness” to infected cells compared with uninfected cells in their growth in the epithelial basal layer, which eventually leads to elimination of noninfected cells from the lesion (34, 35). Interestingly, we were able to locate two microlesions in these immunocompetent mice (Fig. 2). While appearing briefly productive at both of these sites, no macroscopic lesions formed on the tail, which was in agreement with previous research (36). We can infer from their rare occurrence rates compared to the immunodeficient model that these microlesions occur transiently before clearance by the immune system. Or they may occur at such small, controlled sites that they are simply extremely hard to locate. In previous reports, MmuPV1 is shown to form macroscopic lesions in UVB-irradiated or T-cell-depleted immunocompetent mice, suggesting that T-cell-mediated immunity is controlling macroscopic lesion formation (12, 36). In NOD/SCID mice, which have a deficiency in T, B, and NK cells, MmuPV1 showed only minimal disease at cutaneous sites, but developed persistent infection at the mucosal sites, including those of the anogenital region and the oral cavity, suggesting MmuPV1 may have tissue (mucosal) preference (37). Indeed, MmuPV1 persistent and productive lesions have been located in the reproductive tract of FVB immunocompetent mice (8). As such, the phenotype of MmuPV1 infection (incubation time, macroscopic/microscopic lesion formation, clearance, persistent infection, or pattern of viral gene expression) also appears to be determined by co-cofactors (e.g., host/local immunity, genetic backgrounds, inflammation, or site of infection).

The viral genes of papillomaviruses are categorized as core or accessory genes (14). In general, the viral core genes carry out essential functions during the virus life cycle in the epithelium, and these basic functions are conserved throughout papillomaviruses. L1 encodes the primary structural protein in the virus capsid, with the minor capsid protein L2 binding to the circular viral DNA to facilitate optimal genome encapsidation. E1 encodes a virus-specific DNA helicase, while E2 functions in viral transcription, replication, and genome partitioning. In contrast, the accessory genes encode proteins that modify the cellular environment. In many cases, these proteins perform similar but not necessarily identical functions during the life cycle of different papillomaviruses to support lesion formation/maintenance, and the production of progeny virions, which are thought to determine their pathogenicity. During the early steps of MmuPV1 lesion formation, papillomavirus gene expression cellular phenotypes, such as increased cellular density and delayed differentiation (Fig. 1, 2, and 4), were observed, suggesting that the infected cells have a growth advantage or remain more preferentially in the basal layer compared to adjacent noninfected cells. As the expression level of MmuPV1 accessory genes (E6 and E7) showed some heterogeneity and the higher expression may correlate to a cellular morphology which can be characterized as cells leaving the basal layer (delamination), virus genes might also be relevant to the delamination step of infected cells (Fig. 4), but this should be elucidated in future study. Results from keratinocytes expressing MmuPV1 E6 or E7 genes showed that MmuPV1 E6 was responsible for these cellular phenotypes, not MmuPV1 E7. MmuPV1 E6 appeared to inhibit contact inhibition, increase growth postconfluence, resulting in higher saturation density, and delay keratinocyte differentiation (Fig. 6). This is similar to phenotypes observed in our recently published article, which showed exogenous expression of low-risk HPV11 E6, but not E7, afforded a growth advantage and differentiation delay to keratinocytes postconfluence (38), suggesting there may be similar mechanisms between mouse papillomavirus and low-risk HPV types, despite their relatively distant relationship (39). In the competition assay, which allows us to investigate the growth advantages of certain types of cells in mixed-cell populations in 3D by isolating discrete layers of cells within the monolayer culture, keratinocytes expressing MmuPV1 E6 demonstrated a clear phenotype of persistence in the “lower” layer of cells when cultured with control keratinocytes, whereas expression of MmuPV1 E7 did not (Fig. 7). These data suggest that expression of key virus proteins in distinct locations of the cell monolayer, and so possibly within the tissue, affords a cellular fitness over uninfected cells in the basal layer. Overall, our results suggest that the primary functions of E6 in the viral life cycle is to maintain the infected cells in the basal layer and to support lesion formation. It is plausible to suggest that E7 could function in the upper layer of infected epithelial cells by driving cell cycle entry to produce pseudo-S phase and supporting viral genome amplification, which must be investigated in future studies. The main limitation of our *in vitro* experimental model is the use of a spontaneously immortalized human keratinocyte line (NIKS) for evaluation of mouse papillomavirus gene function. The use of NIKS has also some advantages. The isogeneity as well as non-virus-gene-dependent growth of NIKS allow us to conduct comparative analysis and reproduce the data more consistently. The increased fitness of cells in the propagation in the basal layer was confirmed in the competition assay using mouse primary tail keratinocytes (data not shown); however, further investigations should be done to elucidate how papillomavirus genes of each PV have evolved their functions to maximize the fitness to each site of infection (keratinocytes of the different sites of body) in future.

In the past 2 decades, signaling pathways involved in the control of keratinocyte behavior relating to epithelial tissue homeostasis have been described (40, 41). However, knowledge of the molecular mechanisms by which cells respond to a given signal, such as cell density, mechanical stress, and growth factors, is insufficient to explain how a specific cell might change its behavior (proliferation and commitment of differentiation) and how other surrounding cells behave differently in response.

Notch signaling mediates short-range signaling interactions between a cell and its neighboring cells and controls the fate of each differently. In doing so, Notch is thought to be a master regulator of keratinocyte differentiation. In normal stratified epithelium, the activity of the Notch pathway is spatially regulated by the preferential distribution of Notch ligands and receptors in the different epidermal layers. The basal layer mainly expresses Notch ligands: in contrast, the Notch receptors are enriched in the suprabasal layers where Notch signaling is considered to be most active (42, 43). The Notch pathway is known to be required for the transition of keratinocytes from the basal to the suprabasal layers (44). Several PV E6 proteins are also known to target Notch pathways. High-risk HPV types appear to downregulate the expression of Notch receptor via the degradation of p53 (45), which results in repression of differentiation markers in keratinocytes (24, 45). In contrast, cutaneous low-risk HPV8 and MmuPV1 E6 proteins do not target p53 or Notch directly, but bind MAML1, a transcriptional coactivator in the Notch complex, to inhibit Notch activity (25). Indeed, in our study the MmuPV1-E6^R130A^ mutant, which cannot bind to MAML1, failed to show phenotypes such as higher saturation density, inhibition of contact-inhibition/differentiation, or an advantage in the high-density competition assay. In contrast, all of these phenotypes are observed with wild-type MmuPV1 E6 expression, supporting the idea that the function of MmuPV E6 in the viral life cycle is to give the infected cells a competitive advantage over normal cells primarily by the downregulation of the Notch signaling pathway. Recently published work generated a mouse model carrying an inducible dominant-negative mutant of MAML1, which results in inhibition of NICD-induced transcription in the esophageal keratinocyte cell populations (46). The mutant cells dramatically outcompeted their normal counterparts over time *in vivo.* The persistence of these mutant cells was shown to be due to the MAML1 binding-deficient cells not being lost from the basal layer by differentiation and by their stimulating normal neighbor cells to differentiate. These mutant cell populations also exhibited an increased cell density at 30% confluence, characterized by a resulting buckling of the epithelium (47). Interestingly, we found that HES1 expression, a downstream target of Notch signaling, was not downregulated in MmuPV lesions and was in fact significantly higher in the basal cells of early lesions compared to uninfected epithelium (Fig. 5). The lesion also appeared to have delayed differentiation where higher HES1 RNA levels were observed. Recent research into the Notch signaling pathway has found that discrete ligand activation of the pathway can have distinct downstream effects. Importantly, it was found that Dll1 and Dll4, two Notch ligands, modulated the Notch receptor in either short frequency-modulated pulses or sustained amplitude-modulated signals, respectively. Notch pathway activation by Dll1 led to upregulation of Hes1, while Dll4 signaling led to upregulation of Hey1 and HeyL (48). Therefore, we cannot consider Hes1 to be directly representative of a Notch-directed differentiation phenotype in tissue; it appears two discrete pathways can be activated through the Notch receptor. Thorough research into the involvement of Notch in epidermal cell fate has distilled discrete modes of action that Notch signaling activates in spinous cells. First, there can be upregulation of genes required for suprabasal cell differentiation, along with downregulation of genes required to be expressed in the basal layer. In addition, Notch directs maintenance of a proliferative cell phenotype, but can also direct initiation of terminal differentiation. This research showed that Hes1 may in fact be required for maintenance of a proliferative phenotype and that promotion of differentiation occurs in a Hes1-independent manner (49). It is obvious from our data that the activation of HES1 results from stimulation of a proliferative phenotype in the cells that are also positive for E6/E7 RNA expression. An earlier article also indicated that upregulation of Hes2 and Hey1 was dependent on a p63-mediated downregulation of Hes1, demonstrating p63 modulation of Notch-dependent transcription (50). Overall, it seems that HES1 expression in the tissue is indicative of Notch activation; however, this is not necessarily a differentiation phenotype and is much more complex than originally thought.

The Hippo pathway regulates two transcription factors, YAP/TAZ, and play a central role of sensing the physical and mechanical properties of the microenvironment (cell to cell, and cell to extracellular matrix [ECM]) (51). Hippo also plays a critical role in a wide range of biological processes, including organ size control, cell proliferation, cancer development, and virus-induced diseases (reviewed in reference 52). The involvement of the Hippo pathway in the papillomavirus life cycle or pathogenesis has been recently highlighted but little investigated, with literature providing some evidence of it; high-risk HPV E6 and HPV8 E6 seem to be targeting and downregulating the Hippo pathway (53, 54), and the cross talk between YAP/TAZ and the Notch signaling pathway has been reported (41). Further research into this interplay of pathways in the context of virus infection is required to explain the molecular mechanisms of papillomavirus pathogenicity.

This work provides evidence of a role for mouse papillomavirus E6 protein in allowing competitive persistence of infected cells at a higher density in the lower layer of monolayer culture, likely mimicking the dynamics of homeostasis in stratified epithelium, especially in the basal layer *in vivo*. MmuPV1 E6 plays a key role, through regulation of the Notch signaling pathway, in the ability of single infected cells to persist in the basal layer of the epithelium over time to allow the establishment of a productive lesion. Disruption of this competitive advantage of lower-layer persistence could stimulate the detachment, differentiation, and subsequent loss of the infected reservoir of cells, providing a mechanism by which such low-level infection could be treated. If similar mechanisms of persistence are present in papillomaviruses that can cause human disease, therapeutics targeting this pathway could be utilized in tandem with established treatment methods aiming to surgically remove infected cells.

## MATERIALS AND METHODS

### Cell culture.

Normal immortalized keratinocytes (NIKS) (a gift from Paul Lambert, McArdle Laboratory for Cancer Research, University of Wisconsin) were maintained in FC medium with γ-irradiated J2-3T3 cells as previously described (38, 55). Mouse primary keratinocytes were isolated from the tail of a nude mouse using Dispase II (Sigma) and trypsin and then were maintained in FC medium with10 μM ROCK inhibitor (Y-27632; Generon).

### Vector construction and retroviral infection.

Recombinant retroviruses were produced, and transduction carried out as previously described (56). Construction of the retroviral vectors LXSN-MmuPV1E6 and MmuPV1E7 was accomplished by cloning the coding sequences utilizing Gateway Technology (Thermo Fisher Scientific, MA, USA) following manufacturer’s instructions. The LXSN-MmuPV1E6^R130A^ mutant was generated using KOD-Plus- mutagenesis kit (Toyobo, Japan) using primers (AGGCTACTGCGGGTTCTGC and GCCCACATGTGGCGCACC). All constructs generated were sequenced to ensure no additional base changes had occurred. QCXIP-eGFP and QCXIP-mCherry vectors were a gift from Tohru Kiyono, National Cancer Centre, Japan. After transduction, NIKS cells expressing eGFP and mCherry were sorted into four quartiles based on fluorescent intensity using the BD FACSAria fusion cell sorter.

### Growth and high-density competition assays.

In order to represent the growth conditions of the basal layer of stratified epithelium in a 2D *in vitro* assay, cells were seeded at high (confluent) density in a 4-well imaging chamber (MoBiTec, Germany). To each chamber, 2 × 10^5^ cells each of the NIKS experimental mCherry and eGFP groups were seeded with 4 × 10^4^ irradiated J2-3T3 feeder cells. Cells were cultured for up to 10 days, changing the medium every other day, before being fixed in paraformaldehyde (PFA) for 10 min. Cells were visualized by confocal microscopy (LSM 700; Zeiss, Germany).

### SDS-PAGE and Western blotting.

Proteins were extracted from cells using radioimmunoprecipitation assay (RIPA) buffer and quantified using the bicinchoninic acid (BCA) protein assay kit (Pierce, MA, USA), before being separated on 4 to 12% gradient polyacrylamide SDS-Tris-Tricine denaturing gel (Invitrogen) and transferred onto polyvinylidene difluoride (PVDF) membranes (Bio-Rad). After transfer, membranes were blocked for 1 h at room temperature in 1% milk in PBS-T (phosphate-buffered saline plus 0.1% Tween 20). Blots were then incubated overnight at 4°C with the appropriate primary antibody diluted in 1% milk in PBS-T. The primary antibodies used were anti-GAPDH (anti-glyceraldehyde-3-phosphate dehydrogenase) (clone MAB374; Millipore, MA, USA), anti-MAML1 (clone D3E9; Cell Signaling Technology, MA, USA), and anti-hemagglutinin (anti-HA) (clone ab130275; Abcam, Cambridge, United Kingdom). Membranes were then incubated with IR dye (800CW) fluorescent secondary antibody (LI-COR, NE, USA) for 1 h at room temperature and then washed. Finally, proteins were detected using the Odyssey imaging system (LI-COR, NE, USA).

### MmuPV1 preparation.

Mus musculus papillomavirus 1 (MmuPV1) was extracted from mouse lesions as previously described (29). Tissue homogenates and cell lysates were incubated at 37°C with Benzonase for 24 h. After a low-speed centrifugation step to remove cellular debris, the virus particles were pelleted by ultracentrifugation then resuspended with PBS plus 10% fetal bovine serum (FBS) (cell-free virus) (57). Encapsidated virus genome copy number (viral gene equivalent [VGE]) was determined by SYBR green quantitative PCR (qPCR) (Thermo Fisher Scientific, MA, USA) using type-specific primers (GGTGAGCCTGACCTACCCGA and CGGAGAACAGTGTCGCAGCA).

### Animal work and ethics.

All animal procedures were conducted in accordance with the Animals (Scientific Procedures) Act of 1986. The protocols were approved by the Animal Welfare and Ethical Review Body (AWERB) of the University of Cambridge and the Home Office (project license no. 70/8113). Tails of athymic nude mice (Hsd:Athymic Nude-Foxn1^nu^, female, 6 to 8 weeks of age, after a week of acclimatization; Envigo, Indianapolis, IN, USA) or B6 mice (C57BL/6JOlaHsd, female, 6 to 8 weeks of age, after a week of acclimatization; Envigo) were inoculated with 2 × 10^8^ (in a 2-μL volume) cell-free MmuPV1 following a 3-mm-long scarification of the epidermis (up to 3 inoculation sites per tail). Mock infections were carried out with PBS. Tails were harvested after each observation period. Mice were injected intraperitoneally with 200 μL of a 10-mg/mL BrdU (Sigma-Aldrich, United Kingdom) solution 24 h before the harvest. For each experimental group, three slides were selected for analysis. For each slide, 7 fields of view were randomly selected.

### Immunohistochemistry and immunofluorescence.

Immunofluorescence and immunohistochemistry were performed as previously described (58). The formalin-fixed, paraffin-embedded tissue sections were incubated in Target retrieval solution (pH 9) (Dako, Glostrup, Denmark) for 10 min at room temperature prior to incubation for 15 min at 110°C. The cells were washed in PBS and fixed in 4% paraformaldehyde (PFA) in PBS for 10 min at room temperature. The cells were permeabilized in PBS with 0.1% Triton X-100 (Promega) for 30 min, then washed in PBS. The sections and cells were blocked in 10% normal goat serum in PBS for 1 h prior to incubation with the primary antibodies. The antibodies used were an in-house rabbit anti-MmuPV1 E4 and L1 monoclonal antibodies (29), mouse anti-DE-K10 (Invitrogen, CA), rat anti-BrdU, and rabbit anti-cytokeratin 17 (Abcam, Cambridge, United Kingdom). Antigen-antibody complexes were visualized with an anti-mouse Alexa Fluor 488- or 594-conjugated antibody (Thermo Fisher Scientific). Nuclei were visualized with DAPI. BrdU- and K10- positive cells were counted manually.

### RNA *in situ* hybridization.

Viral transcripts of cells were detected and visualized using the RNAScope in situ hybridization assay (Advanced Cell Diagnostics, MN, USA) following the manufacturer’s instructions. The probe used for MmPV1 RNA detection was MusPV-E6-E7 (catalog no. 409771), and the probe used for HES1 RNA was Mm-Hes1-C2 (catalog no. 417701-C2).
